# Chemotherapy Combined With Recombinant Human Endostatin (Endostar) Significantly Improves the Progression-Free Survival of Stage IV Soft Tissue Sarcomas

**DOI:** 10.3389/fonc.2021.778774

**Published:** 2022-01-03

**Authors:** Zhichao Liao, Chao Zhang, Tielong Yang, Haotian Liu, Songwei Yang, Ting Li, Ruwei Xing, Sheng Teng, Yun Yang, Jun Zhao, Gang Zhao, Xu Bai, Lei Zhu, Jilong Yang

**Affiliations:** ^1^ Department of Bone and Soft Tissue Tumor, Tianjin Medical University Cancer Institute and Hospital, Tianjin, China; ^2^ National Clinical Research Center for Cancer, Key Laboratory of Cancer Prevention and Therapy, Tianjin’s Clinical Research Center for Cancer, Tianjin Medical University Cancer Institute and Hospital, Tianjin, China; ^3^ Departments of Bone and Soft Tissue Tumor, Chongqing University Cancer Hospital, Chongqing, China; ^4^ Department of Pathology, Tianjin Medical University Cancer Institute and Hospital, Tianjin, China; ^5^ Department of Radiology, Tianjin Medical University Cancer Institute and Hospital, Tianjin, China; ^6^ Department of Molecular Imaging and Nuclear Medicine, National Clinical Research Center for Cancer, Tianjin Medical University Cancer Institute and Hospital, Tianjin, China

**Keywords:** sarcoma, endostar, chemotherapy, undifferentiated pleomorphic sarcoma, progression-free survival, adverse events

## Abstract

**Purpose:**

Our previously study showed that recombinant human endostatin (Endostar) combined with chemotherapy had significant activity to increase the mPFS in patients with advanced sarcomas with tolerable side effects. However, the small cohort size and short follow-up time made it difficult to screen sensitive sarcoma subtypes and determine whether there is an overall survival benefit. With the largest sarcoma cohort to our knowledge, we try to confirm the efficacy and safety of chemotherapy combined with Endostar in stage IV sarcomas, with the specific purpose of finding out the sensitive sarcoma types for this combined treatment.

**Methods:**

After the exclusion of ineligible patients, 156 patients with stage IV bone and soft tissue sarcomas were included in this study according to the inclusion criteria.

**Results:**

By the end of follow-up, the ORR was 10.7% (9/84) vs 1.4% (1/72) (p=0.041), the DCR was 26.2% (22/84) vs 5.6% (4/72) (p=0.001) in the combined group and chemotherapy group, respectively. The mPFS of combined group was significantly longer than the chemotherapy group (10.42 vs 6.87 months, p=0.003). The mOS were 26.84 months and 23.56 months, without significant difference (p= 0.481). In osteogenic sarcoma, there was no statistically significant difference in the mPFS between the two groups (p=0.59), while in the soft tissue sarcoma, the mPFS in the combined group was significantly higher than that of the chemotherapy group (11.27 vs 8.05 months, p=0.004). Specifically, undifferentiated polymorphic sarcoma (UPS) was the possible sarcoma subtypes that benefited from the combined therapy. For the 38 UPS patients (28 patients in the combined group and 10 patients in the chemotherapy group), the mPFS in the combined group was up to 14.88 months, while it was only 7.1 months in the chemotherapy group, with a significant difference (p=0.006). The most common adverse events in the combined group were myelosuppression, gastrointestinal reactions and abnormal liver function, without significant difference in two groups.

**Conclusion:**

Chemotherapy plus Endostar could prolong mPFS and improve ORR and DCR in patients with stage IV soft tissue sarcoma, suggesting that the combined therapy could improve the patient prognosis in soft tissue sarcomas, especially the UPS patients.

## Introduction

Sarcomas are highly malignant tumors with complex pathological types and large heterogeneity and are mainly divided into two major categories: osteogenic sarcoma and soft tissue sarcoma, both of which have a poor prognosis (1–3). The median overall survival (mOS) for patients with metastatic soft tissue sarcoma is only 12.8-14.3 months (4). For patients with lung metastases or chemotherapy-resistant osteosarcoma, the 5-year survival rate is only 20% (5, 6).

Currently, chemotherapy remains the cornerstone of treatment for patients with advanced unresectable sarcoma. For patients with soft tissue sarcoma, anthracycline-based combination chemotherapy is the first-line chemotherapy regimen. When first-line chemotherapy fails, the options include gemcitabine, docetaxel, ifosfamide, dacarbazine, etc. (4, 7–10). The standard chemotherapy regimens for osteosarcoma patients include high doses of methotrexate, cisplatin, and ifosfamide (5). Unfortunately, despite advances in surgical techniques, the prognosis of patients with bone and soft tissue sarcoma has not improved significantly in recent years. For example, the OS rate for patients with osteosarcoma, which reached 65%-70% in the 1970s, has not improved significantly over the past three decades and has reached a plateau (11). In recent years, there have been an increasing number of clinical trials evaluating the value of chemotherapy combined with other therapies in patients with sarcoma. The combination of doxorubicin and olaratumab (an inhibitor that blocks the PDGF pathway) significantly prolonged the mOS in a phase II trial but failed to demonstrate this finding in a phase III trial (12, 13). A phase II clinical trial showed that paclitaxel combined with bevacizumab was not recommended for the treatment of angiosarcoma due to increased adverse events (14). The combination of chemotherapy agents, such as doxorubicin combined with trabectedin, also does not show any advantage over doxorubicin alone (15). Doxorubicin combined with immunotherapy, such as pembrolizumab, has shown promising progression-free survival (PFS) and OS benefits, but further study is needed because a small number of patients was enrolled (16). Therefore, there is an urgent need to find new treatments to improve the chemotherapy sensitivity of patients with advanced sarcoma.

The growth and metastasis of malignant tumors are closely related to angiogenesis, so antiangiogenic therapy is always a hot research spot and direction of tumor treatment (17–19). Endostatin is an endogenous protein that can inhibit the expression of vascular endothelial growth factor (VEGF); therefore, tumor angiogenesis can be inhibited (20, 21). However, endostatin is unstable under *in vitro* conditions (11). Endostar is a safe and well-tolerated recombinant human endostatin that can inhibit the growth and metastasis of tumors (22). Previous clinical trials have demonstrated definitive antitumor activity of Endostar in patients with lung cancer, breast cancer, melanoma and nasopharyngeal carcinoma, with manageable and tolerable adverse events (23–30). Several studies of Endostar in combination with chemotherapy for the treatment of advanced sarcoma have shown encouraging results, one of which was performed at our cancer center (11, 31–33). These results suggested that Endostar combined with chemotherapy may be a promising treatment for patients with advanced sarcoma. However, these studies were limited by small sample sizes and did not screen the pathological subtypes sensitive to Endostar combined with chemotherapy. Therefore, in the present study, we enrolled the largest stage IV sarcoma cohort who received Endostar combined with a chemotherapy regimen and attempted to analyze independent risk factors affecting prognosis. In addition, we screened for the pathological subtypes with the greatest sensitivity to Endostar combined with chemotherapy. The results suggested that stage IV soft tissue sarcomas, especially undifferentiated pleomorphic sarcoma (UPS), might benefit from the combination of Endostar with chemotherapy, which might supply more management choices for patients with advanced sarcoma after more evidence accumulations.

## Materials and Methods

### Patients and Treatment

**Figure 1:**
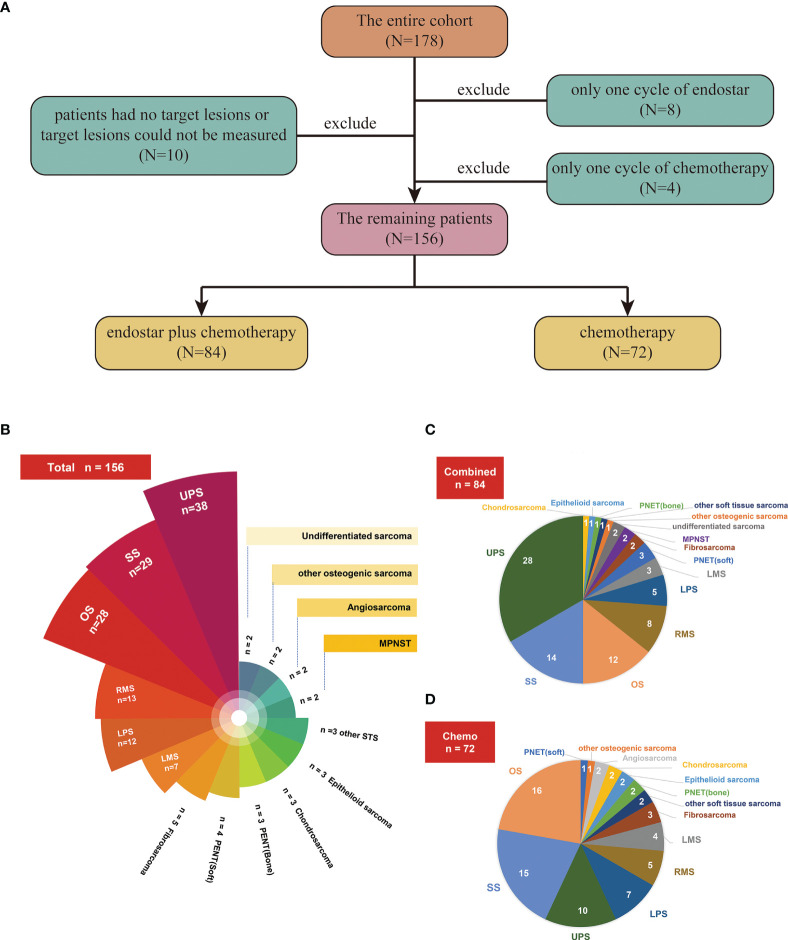
**(A)** Flow chart for screening patients. **(B)** Numbers of patients with different pathological subtypes in the entire cohort. **(C)** Numbers of patients with different pathological subtypes in the Endostar combined with chemotherapy group. **(D)** Numbers of patients with different pathological subtypes in the traditional chemotherapy group.

A total of 178 patients with advanced sarcoma who were admitted to the Department of Bone and Soft Tissue Tumor, Tianjin Medical University Cancer Institute and Hospital, between July 2009 and September 2020 were included in this study (**Figure 1A**). All patients were pathologically confirmed to have bone or soft tissue sarcoma, and the pathological types included the most common types of bone and soft tissue sarcoma. Among the patients, 10 had no target lesions or the target lesions could not be measured, 8 received only one cycle of Endostar treatment, and 4 received only one cycle of chemotherapy. These patients were excluded from the final efficacy and adverse event evaluations (**Figure 1A**). Of the remaining 156 patients, 84 who received Endostar combined with chemotherapy were assigned as the combined group (**Figure 1C**), and 72 patients who received only chemotherapy (**Figure 1D**) were assigned as the chemotherapy (control) group. This study of combination of Endostar and chemotherapy complied with the Declaration of Helsinki and was approved by the Ethics Committee of Tianjin Medical University Cancer Institute and Hospital (Ethical batch number, E2017023).

The chemotherapy regimens used in the two groups mainly included AI, T10, GT, MAID, CAV, and IE. Patients with osteosarcoma received T10 as the main chemotherapy method, while patients with soft tissue sarcoma primarily received AI, MAID, and GT (**Table 1**). In the combined group, 15 mg Endostar (dissolved in 500 ml physiological saline) was given per day during chemotherapy, used continuously for 4 hours for 14 consecutive days, and used again after at least 7 days. Each patient’s heart rate and blood pressure, blood oxygen saturation and skin allergic reactions were closely monitored during the use of Endostar. All patients in this group received at least two cycles of Endostar treatment.

**Table 1:** Patients Characteristics.

	overall	chemotherapy	endostar plus chemotherapy	p value
**patients(%)**	156	72	84	
**gender**				0.382
male	95 (60.9)	47 (65.3)	48 (57.1)	
female	61 (39.1)	25 (34.7)	36 (42.9)	
**age median [range]**	45.50 [10, 81]	44.50 [10, 81]	48.00 [11, 72]	0.828
<60 years	120 (76.9)	55 (76.4)	65 (77.4)	0.883
>=60 years	36 (23.1)	17 (23.6)	19 (22.6)	
**pathological classification**				0.139
osteogenic sarcoma	36 (23.1)	21 (29.2)	15 (17.9)	
soft tissue sarcoma	120 (76.9)	51 (70.8)	69 (82.1)	
**location**				0.227
extremity	111 (71.2)	57 (79.2)	54 (64.3)	
trunk	28 (17.9)	10 (13.9)	18 (21.4)	
retroperitoneum	10 (6.4)	2 (2.8)	8 (9.5)	
head/neck	3 (1.9)	1 (1.4)	2 (2.4)	
others	4 (2.6)	2 (2.8)	2 (2.4)	
**ECOG**				0.146
<=1	100 (64.1)	51 (70.8)	49 (58.3)	
>1	56 (35.9)	21 (29.2)	35 (41.7)	
**surgery**				0.76
wide resection	89 (57.1)	45 (62.5)	44 (52.4)	
amputation	19 (12.2)	8 (11.1)	11 (13.1)	
local resection	26 (16.7)	10 (13.9)	16 (19.0)	
slicer biopsy	11 (7.1)	4 (5.6)	7 (8.3)	
needle aspiration biopsy	11 (7.1)	5 (6.9)	6 (7.1)	
**metastatic location**				0.311
lung	90 (57.7)	41 (56.9)	49 (58.3)	
lung plus other site	21 (13.5)	7 (9.7)	14 (16.7)	
non-lung	45 (28.8)	24 (33.3)	21 (25.0)	
**radiotherapy**				0.880
no	92 (59.0)	42 (58.3)	50 (59.5)	
yes	64 (41.0)	30 (41.7)	34 (40.5)	
**detailed classification of pathology**				N/A
UPS	38 (24.4)	10 (13.9)	28 (33.3)	
synovial sarcoma	29 (18.6)	15 (20.8)	14 (16.7)	
osteosarcoma	28 (17.9)	16 (22.2)	12 (14.3)	
rhabdomyosarcoma	13 (8.3)	5 (6.9)	8 (9.5)	
liposarcoma	12 (7.7)	7 (9.7)	5 (6.0)	
leiomyosarcoma	7 (4.5)	4 (5.6)	3 (3.6)	
fibrosarcoma	5 (3.2)	3 (4.2)	2 (2.4)	
Ewing’s sarcoma of soft tissue/PNET	4 (2.6)	1 (1.4)	3 (3.6)	
chondrosarcoma	3 (1.9)	2 (2.8)	1 (1.2)	
Ewing’s sarcoma of bone/PNET	3 (1.9)	2 (2.8)	1 (1.2)	
epithelioid sarcoma	3 (1.9)	2 (2.8)	1 (1.2)	
other soft tissue sarcoma	3 (1.9)	2 (2.8)	1 (1.2)	
other osteogenic sarcoma	2 (1.3)	1 (1.4)	1 (1.2)	
angiosarcoma	2 (1.3)	2 (2.8)	0 (0.0)	
malignant peripheral nerve sheath tumor	2 (1.3)	0 (0.0)	2 (2.4)	
undifferentiated sarcoma	2 (1.3)	0 (0.0)	2 (2.4)	
**chemotherapy regimens**				0.082
AD	2 (1.3)	2 (2.8)	0 (0.0)	
AI	72 (46.2)	28 (38.9)	44 (52.4)	
CAV/IE	12 (7.7)	5 (6.9)	7 (8.3)	
GT	25 (16.0)	9 (12.5)	16 (19.0)	
MAID	21 (13.5)	14 (19.4)	7 (8.3)	
T10	23 (14.7)	13 (18.1)	10 (11.9)	
TA	1 (0.6)	1 (1.4)	0 (0.0)	

N/A, Not Available.

### Efficacy Evaluation

Both short-term efficacy and long-term efficacy were evaluated. Short-term efficacy was mainly evaluated at 12 weeks, and the evaluation indexes included complete response (CR), partial response (PR), stable disease (SD), progressive disease (PD), the objective response rate (ORR) and the disease control rate (DCR). Long-term efficacy was evaluated at the end of follow-up, and the indexes included CR, PR, SD, PD, the ORR, the DCR, the median progression-free survival (mPFS) and the mOS. The ORR was calculated as (CR +PR)/total cases × 100%. The DCR was calculated as (CR +PR + SD)/total cases × 100%. PFS was defined as the time from the start of treatment to disease progression; OS was defined as the time from the start of treatment to death from any cause.

### Safety and Toxicity Assessments

156 patients underwent efficacy and safety assessments. Treatment-related adverse events were assessed and graded based on the National Cancer Institute Common Terminology Criteria for Adverse Events (CTCAE, version 5.0).

### Statistical Analysis

All data were analyzed using SPSS 22.0. Chi-square test was used to compare clinical pathologic features, ORR, DCR and adverse events of the two groups.

PFS and OS were calculated by the life table method. Kaplan-Meier and Cox analyses were used to compare PFS between the Endostar combined with chemotherapy and traditional chemotherapy groups, and a p value<0.05 was considered statistically significant.

## Results

### Patient Demographics

All 156 patients (**Figure 1B**) had stage IV sarcoma according to the American Joint Committee on Cancer (AJCC) 8th edition staging system. According to the treatment methods, the patients were divided into two groups: 84 were included in the Endostar combined with chemotherapy group (combined group) (**Figure 1C**), and 72 were included in the traditional chemotherapy group (control group) (**Figure 1D**). In this study, there were 95 males and 61 females, with a median age of 45.50 years (range, 10-81 years). The most common pathological types were UPS (n=38), synovial sarcoma (n=29), osteosarcoma (n=28), rhabdomyosarcoma (n=13), and liposarcoma (n=12), etc. (**Figure 1B** and **Table 1**). There was no significant difference in age, sex, location, pathological classification, chemotherapy regimens, Eastern Cooperative Oncology Group (ECOG) performance status, previous surgery type, previous radiotherapy or metastatic site between these two groups (all p>0.05) (**Table 1**).

### Combination Therapy Significantly Improved the ORR

**Figure 2:**
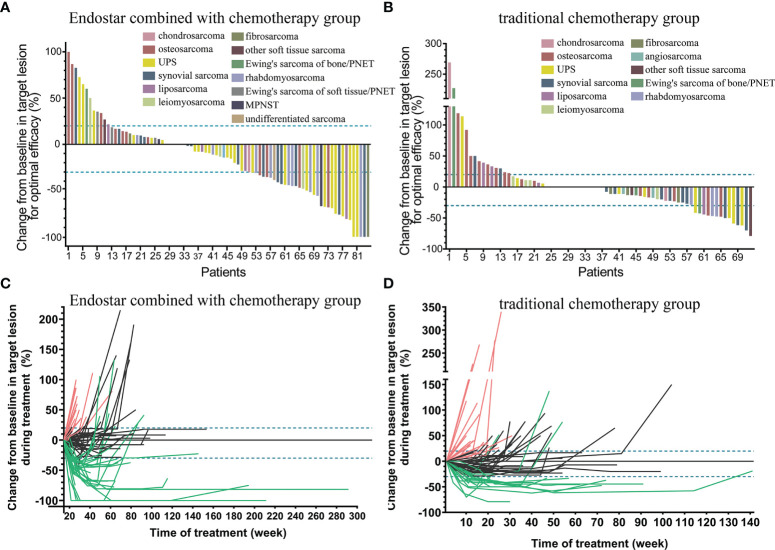
Changes in the maximum diameter of the target lesion in the Endostar combined with chemotherapy group **(A)** and traditional chemotherapy group **(B)** (for optimal efficacy). Continuous changes in the target lesions in the Endostar combined with chemotherapy group **(C)** and traditional chemotherapy group **(D)** during treatment.

Target lesions were evaluated according to the RECIST 1.1. Changes in the maximum diameter of the target lesions in the Endostar combined with chemotherapy group and traditional chemotherapy group (for optimal efficacy) were shown in **Figures 2A, B**. Also the changes in the target lesions in the two groups during treatment are shown in **Figures 2C, D**.

In terms of short-term efficacy, we found obvious differences between the two groups. At 12th week, all patients received at least two cycles of Endostar combined with chemotherapy or chemotherapy alone. Of the 84 patients in the combined group, 5 (5.9%, 5/84) achieved CR, 29 (34.5%, 29/84) achieved PR, 33 (39.3%, 33/84) achieved SD and 17 (20.2%, 17/84) achieved PD. The 12-week ORR was 40.5% (34/84), and the DCR was 79.8% (67/84). Among the 72 patients in the chemotherapy group, no patients achieved CR (0%, 0/72), 13 (18.1%, 13/72) achieved PR, 40 (55.6%, 40/72) achieved SD, and 19 (26.4%, 19/72) achieved PD. The 12-week ORR was 18.1% (13/72), and the DCR was 73.6% (53/72) (**Table 2**). The ORR in the combined group at week 12 was significantly higher than that in the chemotherapy group (p=0.002).

**Table 2:** The efficacy in chemotherapy and endostar combined with chemotherapy.

Efficacy at the 12th week			
	Chemotherapy	endostar combined with chemotherapy	p value
Patients number	72	84	
CR	0/72, 0%	5/84, 5.9%	NA
PR	13/72, 18.1%	29/84, 34.5%	0.021
SD	40/72, 55.6%	33/84, 39.3%	0.042
PD	19/72, 26.4%	17/84, 20.2%	0.363
ORR	18.1% (13/72)	40.5% (34/84)	0.002
DCR	73.6% (53/72)	79.8% (67/84)	0.363
**Efficacy at the end of follow-up**			
	Chemotherapy	endostar combined with chemotherapy	
Patients number	72	84	
CR	0/72, 0%	4/84, 4.8%	NA
PR	1/72, 1.4%	5/84, 5.9%	0.289
SD	3/72, 4.2%	13/84, 15.5%	0.02
PD	68/72, 94.4%	62/84, 73.8%	0.001
ORR	1.4% (1/72)	10.7% (9/84)	0.041
DCR	5.6% (4/72)	26.2% (22/84)	0.001
mPFS (month)	6.87	10.42	0.003
mOS (month)	23.56	26.84	0.481

N/A, Not Available.

Given the fact that chemotherapy and targeted therapy would have secondary resistance and disease progression after multiple cycles, we especially paid attention to the best ORR/DCR and the final ORR/DCR. During the following observation, we noticed that there were some patients who suffered from disease progression both groups, suggesting that the ORR/DCR in the 12th week might be the best ORR/DCR in the both groups in this study.

### Combination Therapy Significantly Improved the mPFS of Stage IV Soft Tissue Sarcoma Patients

**Figure 3:**
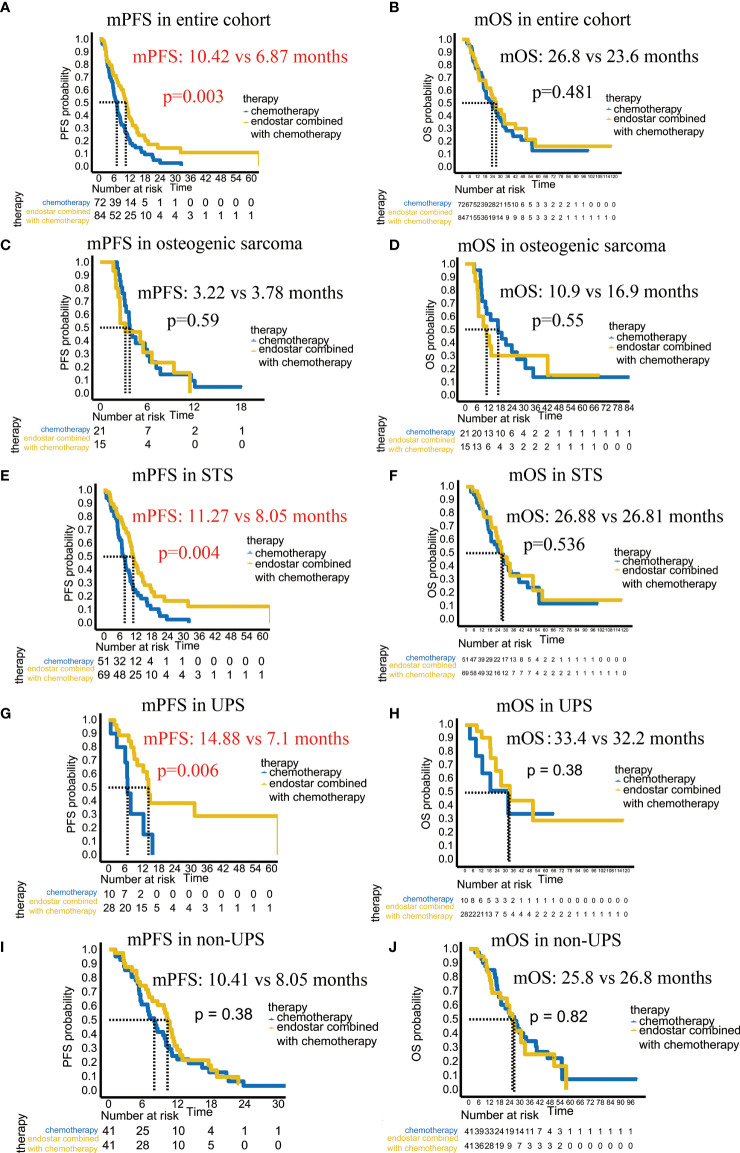
Comparison of the mPFS and mOS between the Endostar combined chemotherapy group and chemotherapy group. **(A)** Comparison of the mPFS in the entire cohort. **(B)** Comparison of the mOS in the entire cohort. **(C)** Comparison of the mPFS in patients with osteogenic sarcoma. **(D)** Comparison of the mOS in patients with osteogenic sarcoma. **(E)** Comparison of the mPFS in patients with soft tissue sarcoma. **(F)** Comparison of the mOS in patients with soft tissue sarcoma. **(G)** Comparison of the mPFS in patients with UPS. **(H)** Comparison of the mOS in patients with UPS. **(I)** Comparison of the mPFS in patients with non-UPS. **(J)** Comparison of the mOS in patients with non-UPS.

The median follow-up period was 16.9 months, ranged from 2.5 months to 118.1 months. By the end of follow-up, the ORR was 10.7%, the DCR was 26.2%, the mPFS reached 10.42 months, and the mOS was 26.84 months in the combined group. In the chemotherapy group, the ORR was 1.4%, the DCR was 5.6%, the mPFS was 6.87 months, and the mOS was 23.56 months. The ORR and DCR of the combined group were significantly higher than those of the chemotherapy group (**Table 2**). Most importantly, there was a significant difference in the mPFS between the combined group and chemotherapy group (10.42 months VS 6.87 moths; p =0.003) (**Figure 3A**). However, there was no significant difference in the mOS (p=0.481) (**Figure 3B**).

**Figure 4:**
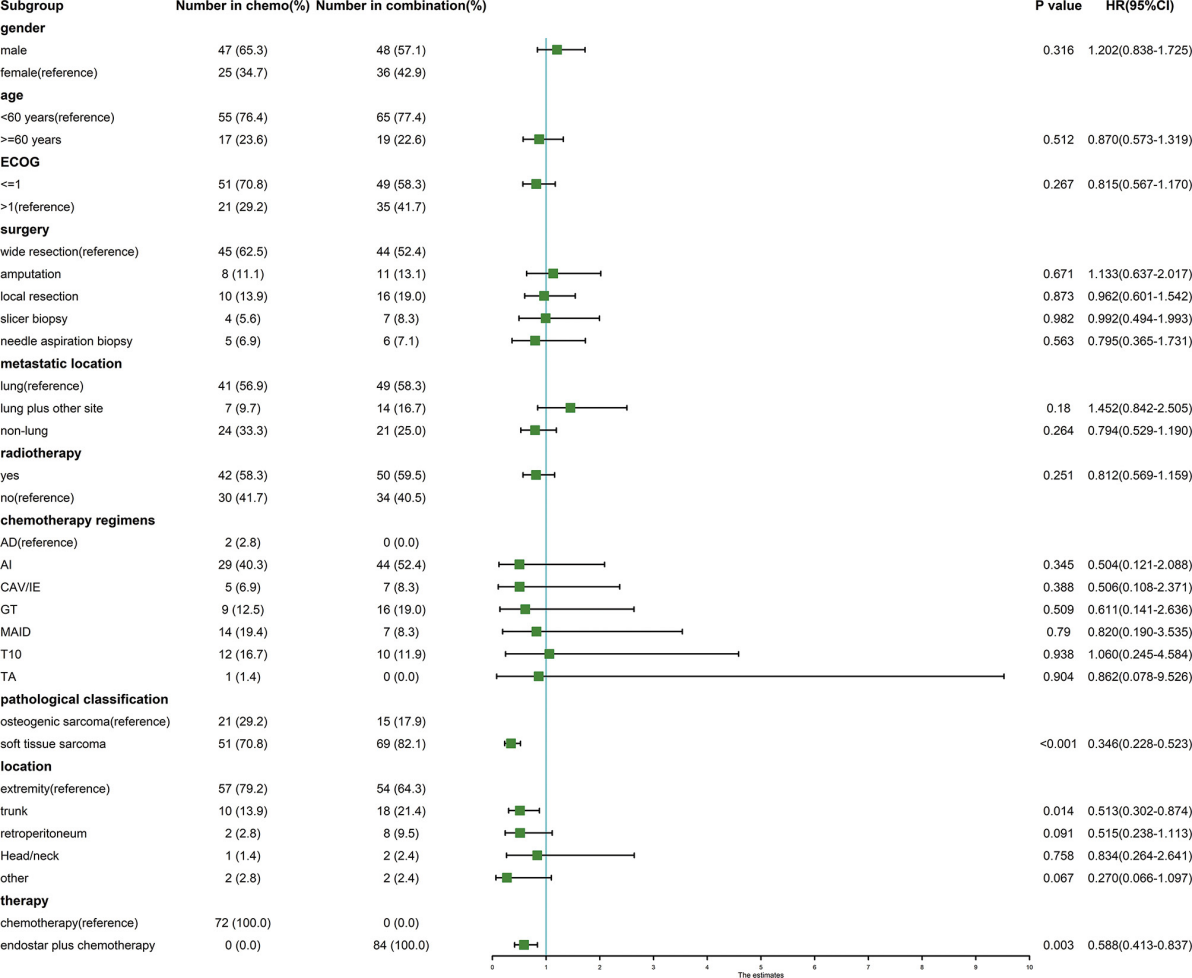
Forest plot of clinicopathological features affecting the mPFS in the univariate Cox analysis.

We analyzed the clinical and pathological features affecting the mPFS. In the univariate Cox analysis, only the pathological classification, location, and combined Endostar to chemotherapy were associated with the mPFS (**Figure 4**). However, in the multivariate Cox analysis, only the pathological sarcoma subtypes (soft tissue sarcoma vs osteogenic sarcoma, p<0.001, HR: 0.383, 95% CI: 0.251-0585) and therapy regiment (Endostar plus chemotherapy vs chemotherapy, p=0.033, HR: 0.675, 95% CI: 0.470-0.969) were associated with the improved mPFS.

We then tried to find out which sarcoma subtype could benefit from the combined therapy with improved mPFS. Our study included 36 patients with osteogenic sarcomas and 120 patients with soft tissue sarcomas. In the osteogenic sarcoma group, the mPFS of the combined group and chemotherapy group were 3.22 months vs. 3.78 months (p=0.59), and the mOS were 10.94 months vs. 16.85 months (p=0.55), respectively, showing no significant improvement (**Figures 3C, D**). For the soft tissue sarcoma patients, the mPFS of the combined therapy group was significantly improved (11.27 months vs 8.05 months, p=0.004), even the mOS (26.88 months vs 26.81 months, p=0.536) showed no difference between these two groups (**Figures 3E, F**). It suggested that the soft tissue sarcomas could significantly benefit from the combined therapy and achieve better mPFS.

### UPS Patients Significantly Benefited From Endostar Combined With Chemotherapy With Improved PFS

Due to the fact that it is the soft tissue sarcoma patients which were significantly benefited from the combined therapy, we further analyzed which soft tissue sarcoma subtype could benefit from the combined therapy. The results revealed that the patients with UPS achieved the better efficacy of Endostar combined with chemotherapy. In this study, there were 38 patients with UPS: 28 patients in the combined group and 10 patients in the chemotherapy group. In the Cox analysis, the chemotherapy regimen and location did not affect the mPFS, but the addition of Endostar to chemotherapy did affect the mPFS (p =0.01). The mPFS of the combined group was up to 14.88 months, while it was only 7.1 months in the chemotherapy group, with a significant difference (p=0.006) (**Figure 3G**). However, there was no statistically significant difference in the mOS (p=0.38) (**Figures 3G, H**). Among the remaining soft tissue sarcoma subtypes, there was no significant difference in the mPFS (p=0.38) or mOS (p=0.82) between the combined group and the chemotherapy group (**Figures 3I, J**). It suggested that the UPS patients treated with combined therapy could achieve better efficacy with improved mPFS.

Additionally, 4 patients suffered from PD after receiving traditional chemotherapy then transfered to the Endostar combined with chemotherapy treatments. But the number of patients was too small to compare whether there was a difference in the mPFS. The first patient developed lung metastases after receiving traditional chemotherapy and was then treated with Endostar combined with AI. After two cycles of combination treatment, PD was achieved, the final PFS was 2.3 months, and the OS was 10.94 months. The second patient developed lung metastasis after three cycles of AI chemotherapy and then received Endostar combined with GT. The efficacy was evaluated as SD at 12 weeks and PD at the end of follow-up, with a final PFS duration of 19.22 months and an OS duration of 23.92 months. The third patient progressed after receiving MAID and GT chemotherapy, followed by Endostar combined with AI. After 6 cycles of combination treatment, the efficacy was evaluated as PR at 12 weeks and PD at the end of follow-up, with a final PFS of 23.06 months and an OS of 30.55 months. The fourth patient progressed after one cycle of treatment with ifosfamide plus liposomal paclitaxel and progressed again after two cycles of treatment with ifosfamide plus liposomal doxorubicin and Endostar, with a final PFS of 2.5 months and an OS of 4.7 months.

### Combination Therapy Might Increase Chemotherapy Sensitivity and Help Achieve CR in Some Soft Tissue Sarcoma Patients

**Figure 5:**
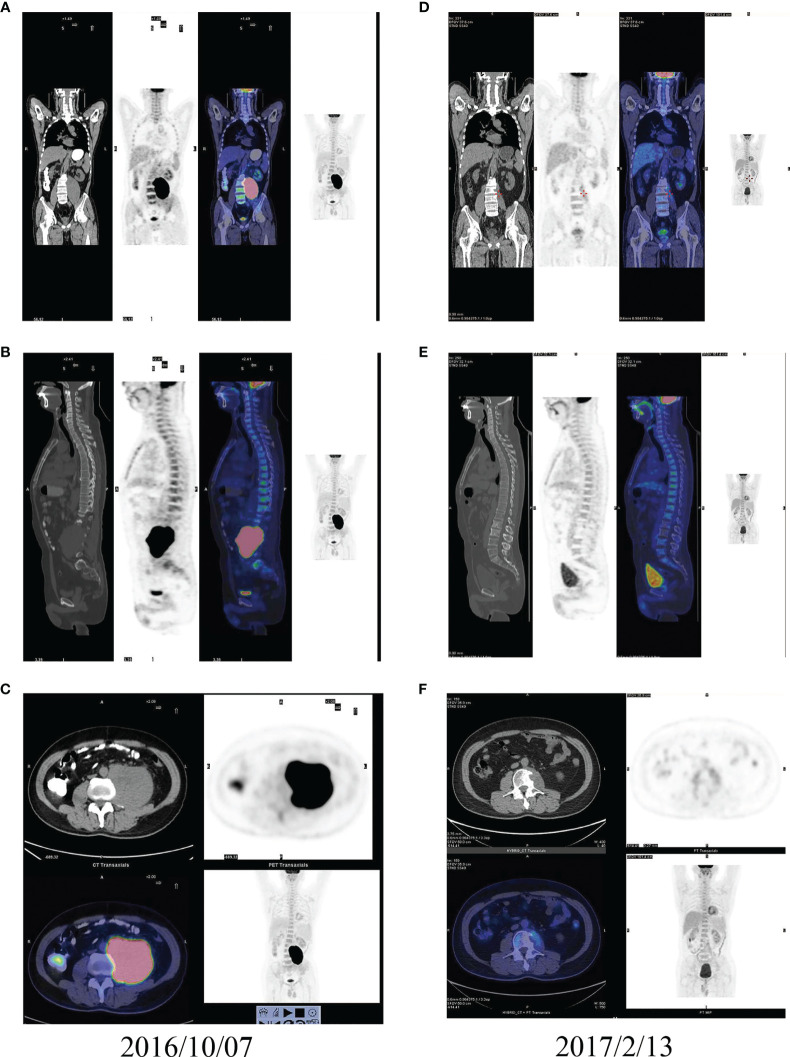
PET-CT comparison of a typical patient with a large retroperitoneal UPS who achieved CR before and after treatment with Endostar combined with AI. **(A)** Coronal PET-CT image before treatment. **(B)** Sagittal PET-CT image before treatment. **(C)** Cross-sectional PET-CT image before treatment. **(D)** Coronal PET-CT image after treatment. **(E)** Sagittal PET-CT image after treatment. **(F)** Cross-sectional PET-CT image after treatment.

**Figure 6:**
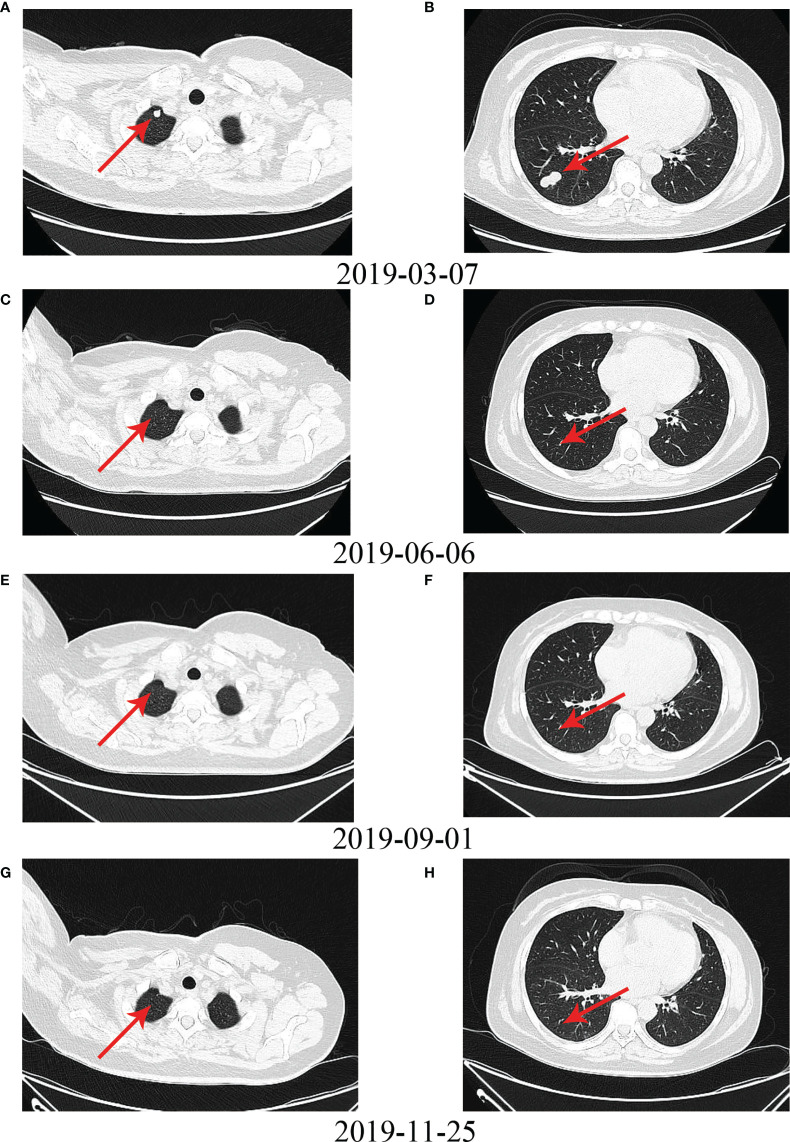
Another typical patient with pulmonary metastases who achieved CR after surgery for thigh synovial sarcoma before and after treatment with Endostar combined with chemotherapy. **(A)** Upper lobe metastases before treatment. **(B)** Lower lobe metastases before treatment. **(C–H)** Metastases in the upper and lower lobes disappeared after treatment with Endostar combined with chemotherapy.

No patient achieved CR in the chemotherapy group, but four patients achieved CR in the Endostar combined with chemotherapy group. **Figures 5A–F** shows the PET-CT comparison of a typical patient who achieved CR before and after treatment with Endostar combined with AI. This was a UPS patient with a large retroperitoneal tumor. Pretreatment imaging showed a large retroperitoneal mass, approximately 13.5 cm in diameter, with significant invasion into the lumbar spine (**Figures 5A–C**). The patient was continuous in pain and could not lie on his back. After treatment with 4 cycles of Endostar combined with AI, the retroperitoneal tumor and lumbar spine lesion disappeared, PET-CT suggested no concentration aggregation, and the efficacy was evaluated as CR (**Figures 5D–F**). **Figure 6** shows another typical patient who achieved CR before and after treatment with Endostar combined with chemotherapy. This was a patient who developed pulmonary metastases after surgery of the thigh synovial sarcoma. After 6 cycles of Endostar combined with AI, the target lesion in the right lung had completely disappeared on CT (**Figures 6A–H**).

**Figure 7:**
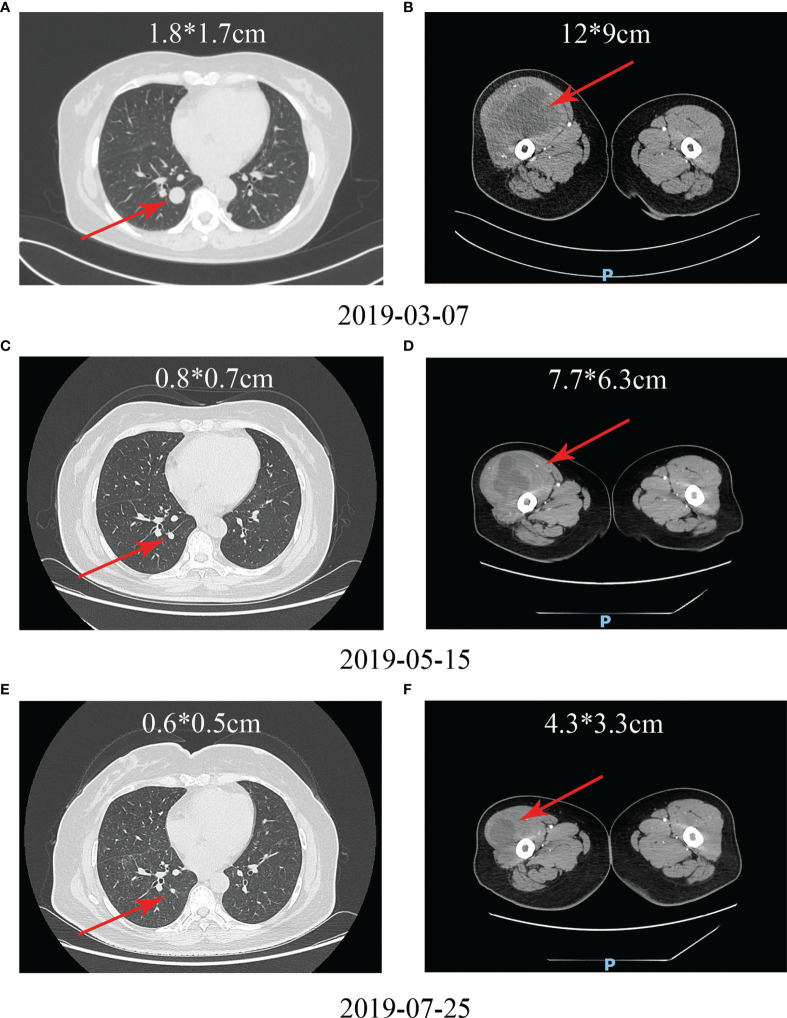
A typical patient with a malignant peripheral nerve sheath tumor in the right thigh and metastases in both lungs detected at the initial consultation who achieved PR. **(A)** Lung metastases before treatment. **(B)** Primary tumor of the right thigh before treatment. **(C–F)** After receiving Endostar combined with chemotherapy, both the lung metastases and the primary tumor of the right thigh were reduced.

In addition to the four patients achieved CR, there were five patients who achieved PR in the Endostar combined with chemotherapy group. MPNST is considered a typical chemotherapy-resistant sarcoma type. The combination of chemotherapy with Endostar significantly increased chemotherapy sensitivity. A patient with MPNST in the right thigh had metastases in both lungs detected at the initial consultation. After giving the patient 4 cycles of Endostar combined with AI, imaging showed a significant reduction in both the primary lesion and lung metastases in the right thigh (**Figures 7A–F**). The patient was treated with palliative surgery and then received maintenance therapy.

### Endostar Combined With Chemotherapy Did Not Increase the Frequency or Severity of Adverse Events Compared With Traditional Chemotherapy

In this study, the majority of adverse events in the combined group were mild (grades 1-2) and manageable. Specifically, grade 1 adverse events accounted for 80% (177/221), grade 2 adverse events accounted for 14% (31/221), grade 3 adverse events accounted for 2.7% (6/221), and grade 4 adverse events accounted for 3.2% (7/221). Myelosuppression was the most common adverse event in the combined group (25.8%, 57/221). Of the patients in this group, seven developed grade IV myelosuppression, and six recovered completely after treatment and continued to receive Endostar combined with chemotherapy; however, the remaining patient discontinued treatment due to adverse events. Other common adverse events included gastrointestinal reactions (24.9%, 55/221), abnormal liver function (23.1%, 51/221), pigmentation (9.5%, 21/221), arrhythmia (9%, 20/221), allergies (5%, 11/221), and renal inadequacy (2.7%, 6/221) (**Table 3**).

**Table 3:** The adverse events in the endostar combined with chemotherapy.

Adverse Event	Grade1	Grade 2	Grade 3	Grade 4	Total
**myelosuppression**	35	11	4	7	57 (57/221, 25.8%)
**gastrointestinal reactions**	53	1	1	0	55 (55/221, 24.9%)
**allergies**	9	1	1	0	11 (11/221, 5%)
**pigmentation**	14	7	0	0	21 (21/221, 9.5%)
**abnormal liver function**	46	5	0	0	51 (51/221, 23.1%)
**arrhythmia**	17	3	0	0	20 (20/221, 9%)
**renal inadequacy**	3	3	0	0	6 (6/221, 2.7%)
**Total**	177	31	6	7	221

Next, we compared the incidence of adverse events between the Endostar combined with chemotherapy group and the traditional chemotherapy group. The results showed no statistically significant difference in the incidence of various adverse events between the two groups (**Table 4**). Furthermore, we compared the incidence of various adverse events between UPS patients and non-UPS patients in soft tissue sarcoma patients who received Endostar combined with chemotherapy. The results showed no significant difference in the incidence of various adverse events between the two groups of patients (**Table 5**). These results suggest that Endostar combined with chemotherapy does not cause additional adverse events compared to chemotherapy alone, and there is no significant difference in the incidence of adverse events for UPS patients.

**Table 4:** Comparison of adverse events between endostar combined with chemotherapy and chemotherapy.

Adverse Event	No. (%) Adverse Events by Treatment		*P*
	control group	combined group	
myelosuppression	54 (54/72, 75%)	57 (57/84, 67.9%)	0.326
gastrointestinal reactions	54 (54/72, 75%)	55 (55/84, 65.5%)	0.196
abnormal liver function	44 (44/72, 61.1%)	51 (51/84, 60.7%)	0.960
pigmentation	10 (10/72, 13.9%)	21 (21/84, 25%)	0.083
arrhythmia	13 (13/72, 18.1%)	20 (20/84, 23.8%)	0.380
allergies	11 (11/72, 15.3%)	11 (11/84, 13.1%)	0.696
renal inadequacy	3 (3/72, 4.2%)	6 (6/84, 7.1%)	0.652

**Table 5:** Comparison of adverse events between UPS and non-UPS in combined group.

Adverse Event	No. (%) Adverse Events by Treatment		*P*
	UPS	non-UPS	
myelosuppression	20 (20/28, 71.4%)	37 (37/56, 66.1%)	0.620
gastrointestinal reactions	19 (19/28, 67.9%)	36 (36/56, 64.3%)	0.746
abnormal liver function	18 (18/28, 64.3%)	33 (33/56, 58.9%)	0.636
pigmentation	5 (5/28, 17.9%)	16 (16/56, 28.6%)	0.285
arrhythmia	6 (6/28, 21.4%)	14 (14/56, 25.0%)	0.717
allergies	3 (3/28, 10.7%)	8 (8/56, 14.3%)	0.909
renal inadequacy	1 (1/28, 3.6%)	5 (5/56, 8.9%)	0.653

## Discussion

Chemotherapy plays an indispensable role in the treatment of stage IV bone and soft tissue sarcoma. Although improvements in surgical procedures have allowed most patients to retain extremity function, overall survival in patients with sarcoma has not improved significantly in recent years (11). Therefore, it is urgent to find a therapeutic method that can effectively improve the chemotherapy sensitivity of patients with tolerable toxicity. In this background, people ushered in the era of antiangiogenic therapy. Endostar, a synthetic recombinant endostatin, has a broad spectrum of antiangiogenic activities mainly by targeting VEGF (22). As early as 2005, Endostar was approved by the China Food and Drug Administration for the treatment of non-small cell lung cancer (34). However, few studies have been conducted in bone and soft tissue sarcoma. Our previous study indicated that Endostar combined with chemotherapy could improve PFS in patients with advanced sarcoma (33). In the present study, we found that the pathological classification and combination treatment were independent risk factors affecting sarcoma patient outcomes. Endostar combined with chemotherapy significantly improved the mPFS of the soft tissue sarcoma patients, especially the UPS. In addition, Endostar combined with chemotherapy did not increase the incidence or severity of adverse events compared to traditional chemotherapy, and the adverse events were tolerable and well controlled.

We believe that this study has the following two advantages. First, this is the largest cohort retrospective study on the efficacy of Endostar combined with chemotherapy in bone and soft tissue sarcoma, with 156 patients enrolled. Due to the rarity of bone and soft tissue sarcoma, few studies to date have evaluated the efficacy and safety of Endostar combined with chemotherapy in bone and soft tissue sarcoma, and most studies have included only a few dozen patients (11, 31–33). One study included 116 patients, but only patients with osteosarcoma were enrolled, and patients with soft tissue sarcoma and other osteogenic sarcoma and soft tissue sarcoma were excluded (11). The small sample size weakened the credibility of these findings to some extent. However, the present study overcame this shortcoming and enrolled 156 patients, which enhanced the credibility of the results. The results showed that Endostar combined with chemotherapy improved patient prognosis at 12th week and at the end of follow-up. At the end of follow-up, Endostar combined with chemotherapy significantly improved the mPFS (10.42 months vs. 6.87 months, p =0.003). These findings are similar to those of two previously reported retrospective studies (11, 32). These results confirmed that Endostar combined with chemotherapy can indeed improve the prognosis of patients. Second, we screened the most sensitive pathological subtypes to Endostar combined with chemotherapy for the first time. Sarcoma is a very heterogeneous malignant tumor with more than 50 pathological subtypes. Whether all pathological subtypes or only specific pathological subtypes are sensitive to Endostar combined with chemotherapy remains unknown. In the present study, the pathological classification and addition of Endostar to chemotherapy were identified as independent risk factors affecting the mPFS. To control for the influence of the pathological classification on the mPFS, we compared the effect of the addition of Endostar to chemotherapy on the mPFS according to different pathological classifications. In osteogenic sarcoma, the presence or absence of Endostar had no effect on the mPFS (p =0.59), while in soft tissue sarcoma, the mPFS of patients treated with Endostar combined with chemotherapy was significantly higher than that of patients treated with traditional chemotherapy (p =0.004). Considering that 12 pathological classifications of soft tissue sarcoma were included in this study, we further screened the pathological classifications. The results showed that the mPFS was significantly higher in UPS patients receiving Endostar combined with chemotherapy (p =0.006). These results suggest that UPS might be the possible pathological subtype that benefits from Endostar combined with chemotherapy. Therefore, Endostar combined with chemotherapy can be applied to improve the prognosis of patients with UPS in future treatment.

In contrast to hand-foot syndrome, hypertension, proteinuria and other common adverse events of antiangiogenic drugs, such as bevacizumab and apatinib, the main adverse events of Endostar were cardiotoxicity, gastrointestinal reactions and allergic reactions (34–38). Adverse events of chemotherapy mainly include myelosuppression, abnormal liver function, gastrointestinal reactions and so on (5, 39). In this study, the major adverse events of Endostar combined with chemotherapy were myelosuppression (25.8%), gastrointestinal reactions (24.9%) and abnormal liver function (23.1%), and the majority of adverse events were grades I and II. Moreover, in terms of the incidence of adverse events, the incidence in the Endostar combined with chemotherapy group was not higher than that in the traditional chemotherapy group. Furthermore, in terms of the only pathological subtype of soft tissue sarcoma that can benefit from endostar combined with chemotherapy, the incidence of various adverse events in UPS patients receiving Endostar combined with chemotherapy was not higher than that in non-UPS patients. Therefore, Endostar combined with chemotherapy did not increase the incidence of adverse events, and these adverse events were controllable and tolerated, indicating that Endostar combined with chemotherapy not only improves the prognosis of patients but is also safe.

Nevertheless, this study still has several drawbacks. First, this was a single-center retrospective study that needs to be validated through a multicenter prospective clinical trial. Second, although the chemotherapy regimen did not affect the mPFS in this study, there were many types of chemotherapy regimens that were not randomly selected, and a randomized, double-blind clinical trial is needed for further validation. Third, in this study, although the number of UPS patients was 38, the number of patients with other pathological types was still relatively small, and the inclusion of other pathological types is needed to further evaluate the efficacy and safety of Endostar combined with chemotherapy in patients with advanced sarcoma. Fourth, previous studies reported that Endostar combined with chemotherapy could improve the mPFS in patients with osteosarcoma (11). However, the results of the present study showed that Endostar combined with chemotherapy could not improve the prognosis of patients with osteosarcoma. The next step is to increase the number of patients with osteogenic sarcoma, including those with osteosarcoma, to further evaluate the efficacy of Endostar combined with chemotherapy.

In conclusion, Endostar combined with chemotherapy significantly improved the mPFS in patients with advanced soft tissue sarcoma, especially the UPS patients, and the adverse events were tolerable. This treatment regimen has shown encouraging objective efficacy and controllable toxicity. Therefore, Endostar combined with chemotherapy can be applied to improve the prognosis of patients with advanced soft tissue sarcoma. In the meantime, more patients need to be recruited, or more rigorous randomized controlled trials need to be conducted to further confirm these findings.

## Data Availability Statement

The raw data supporting the conclusions of this article will be made available by the authors, without undue reservation.

## Ethics Statement

The studies involving human participants were reviewed and approved by Tianjin Medical University Cancer Institute and Hospital Ethics Committee. The patients/participants provided their written informed consent to participate in this study.

## Author Contributions

ZL and CZ drafted and revised the manuscript. CZ and JY sent up the study and corrected the manuscript. ZL, TY, HL, CZ, SY, TL, RX, ST, YY, JZ, XB, and LZ performed experiments and analyzed data. GZ confirmed the pathological diagnosis. JY designed the project. All authors were actively involved in the preparation of this manuscript. All authors contributed to the article and approved the submitted version.

## Funding

This work was partly supported by the Nature Science Foundation of Tianjin (grant number 16JCYBJC24100 to JY and 18YFZCSY00550 to JY).

## Conflict of Interest

The authors declare that the research was conducted in the absence of any commercial or financial relationships that could be construed as a potential conflict of interest.

## Publisher’s Note

All claims expressed in this article are solely those of the authors and do not necessarily represent those of their affiliated organizations, or those of the publisher, the editors and the reviewers. Any product that may be evaluated in this article, or claim that may be made by its manufacturer, is not guaranteed or endorsed by the publisher.

## Abbreviations

UPS, undifferentiated pleomorphic sarcoma; CR, complete response; PR, partial response; SD, stable disease; PD, progressive disease; ORR, objective response rate; DCR, disease control rate; mPFS, median progression-free survival; mOS, median overall survival; AJCC, American Joint Committee on Cancer; MPNST, malignant peripheral nerve sheath tumor; AI, doxorubicin+ ifosfamide; T10, methotrexate+ cisplatin+ doxorubicin; GT, gemcitabine+ paclitaxel; MAID, mesna + doxorubicin+ ifosfamide+ dacarbazine; CAV, cyclophosphamide+ doxorubicin+ vincristine; IE, ifosfamide + etoposide.
